# Triggering final follicular maturation for IVF cycles

**DOI:** 10.1186/s12958-024-01332-5

**Published:** 2025-01-23

**Authors:** Raoul Orvieto

**Affiliations:** 1 Infertility and IVF Unit, Department of Obstetrics and Gynecology, Chaim Sheba Medical Center (Tel Hashomer), Ramat Gan, 52621 Israel; 2 Israel and the Tarnesby-Tarnowski Chair for Family Planning and Fertility Regulation, Faculty of Medical and Health Science, Tel-Aviv University, Tel Aviv, Israel

**Keywords:** hCG, GnRH agonist, Ovulation trigger, OHSS, Controlled ovarian hyperstimulation, Oocyte quality, Dual trigger, Double trigger, Poor responders, High responders

## Abstract

As part of a conventional controlled ovarian hyperstimulation (COH) regimen, final follicular maturation is usually triggered by a single bolus dose of human chorionic gonadotropin (hCG). COH, which combines GnRH antagonist co-treatment with GnRH agonist(GnRHa) trigger, is often used in attempts to eliminate severe early ovarian hyperstimulation syndrome and to improve oocyte/embryo yield and quality. Recently, the combination of GnRHa, with hCG trigger has also been implemented into clinical practice. Here, we analyze and discuss published studies on various ways of triggering final follicular maturation, seeking to elucidate the appropriateness of each approach for specific patient subgroups.

## Introduction

During the ovulatory cycle, sufficient production of estradiol by the preovulatory follicle induces a midcycle surge of luteinizing hormone (LH). This is followed by a loss of gap junctions between the oocyte and cumulus cells, cumulus expansion, germinal vesicle breakdown, resumption of meiosis, and luteinization of the granulosa cells (GCs). The consequent increase in progesterone synthesis facilitates the positive feedback action of estradiol to induce the concomitant midcycle peak of follicle-stimulating hormone (FSH) [1]. This peak FSH has several roles; these include ensuring an adequate complement of LH receptors on the granulosa layer and the synthesis of hyaluronic acid matrix, which facilitates the expansion and dispersion of the cumulus cells, allowing the oocyte-cumulus cell mass to float freely in the antral fluid [1].

## What controls the midcycle LH/FSH peak?

**Fig. 1:**
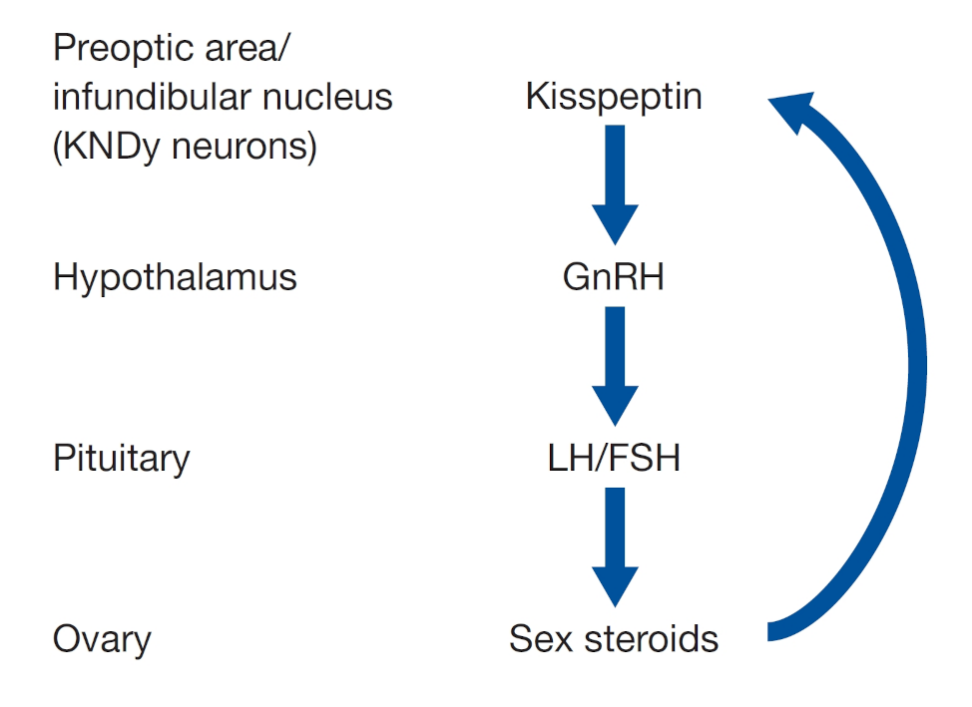
Triggering final follicular maturation: neuroendocrine control

As shown in Fig. 1, kisspeptin neurons, residing within the preoptic area and the infundibular nucleus in the human hypothalamus, co-express neurokinin B and dynorphin (KNDy neurons). Via the neurokinin B and kappa opioid peptide receptors, KNDy neurons autosynaptically regulate secretion of pulsatile kisspeptin, with neurokinin B being stimulatory and dynorphin inhibitory. Kisspeptin signals directly to GnRH neurons in the hypothalamus to secrete gonadotropin-releasing hormone (GnRH), which in turn, is responsible for activating secretion of LH/FSH [2].

## How can we trigger final follicular maturation?

### Human chorionic gonadotropin

As part of a standard/conventional controlled ovarian hyperstimulation (COH) regimen, final follicular maturation is usually triggered by a single bolus dose (5000–10,000 units) of human chorionic gonadotropin (hCG), administered as close as possible to the time of ovulation (36 h before oocyte recovery) [3]. A surrogate to the naturally occurring LH surge, hCG induces luteinization of the GCs, final oocyte maturation, and resumption of meiosis.

Ovarian hyperstimulation syndrome (OHSS) almost always presents either 3–7 days after hCG administration in susceptible patients (“early onset”) or during early pregnancy, 12–17 days after hCG administration (“late onset”). Individualization of treatment based on the specific risk factor and response in the current cycle, with the option of freezing all embryos or replacing just one, can reduce the risk and the severity of OHSS in susceptible cases [4]. Moreover, although withholding the ovulation-inducing hCG trigger has the potential to eliminate severe early OHSS, this approach can be frustrating for the patient and is costly in terms of both time and money.

### Recombinant luteinizing hormone

The European Recombinant Human LH Study Group [5] conducted a prospective, comparative, dose-finding study to compare hCG (5000 IU) with different recombinant human (rhLH) doses (5000, 15,000, 30,000 IU; or 15,000 IU followed 3 days later by 10,000 IU) to induce final follicular maturation in patients undergoing the long GnRH agonist (GnRHa) COH protocol. Although the lowest dose of rhLH (5000 IU) seemed suboptimal compared with the higher dose (15,000 IU), no significant differences were observed between the four doses of rhLH and their corresponding hCG group with regard to the number of oocytes retrieved, number of oocytes retrieved per follicle with a diameter > 10 mm on the day of trigger, oocyte maturation, or number of embryos; nor the number of total, biochemical, and clinical pregnancies, or embryo implantation rate. However, in many of these parameters, in terms of safety, no moderate or severe OHSS was reported in patients who received a single dose of rhLH up to 30,000 IU. The investigators concluded that a single dose of rhLH is effective in inducing final follicular maturation comparable with 5000 IU hCG, with a highly significant reduction in OHSS compared with hCG. Moreover, the dose of rhLH giving the highest efficacy/safety ratio was between 15,000 and 30,000 IU. These observations are promising, but rhLH is expensive to produce.

### GnRH agonist

Controlled ovarian hyperstimulation, combining GnRH antagonist co-treatment and GnRHa trigger, has become standard practice to eliminate the risk of severe early OHSS [6]. However, this approach is reported to result in fewer clinical pregnancies and increased first-trimester pregnancy loss [7]. Measures introduced to improve reproductive outcome include a “freeze-all” policy, fresh transfer and intensive luteal support, and fresh transfer with low-dose hCG supplementation; each of these has its inherent advantages and disadvantages [8].

### Kisspeptin

In 2014, Jayasena et al. [9] published their first dose-finding study of a single subcutaneous injection of kisspeptin-54 (1.6, 3.2, 6.4, and 12.8 nmol/kg) to induce final follicular maturation in patients undergoing the GnRH antagonist COH protocol for in-vitro fertilization (IVF). Egg maturation was observed in response to each tested dose of kisspeptin, with a clinical pregnancy rate of 23%.

Later studies by the same group [10, 11] have demonstrated that the highest oocyte yield was observed following 12.8 nmol/kg kisspeptin, which was 69% greater than following 3.2 nmol/kg. The highest pregnancy rates were observed following 9.6 nmol/kg kisspeptin, with no woman developing moderate or severe OHSS. In women at high risk of OHSS, these studies showed that the effectiveness of a single injection of kisspeptin (9.6 nmol/kg) 36 h before oocyte retrieval was improved by a second dose 10 h later.

A higher proportion of patients achieved an oocyte yield ≥ 60% following a second dose of kisspeptin, without increasing the frequency of ovarian over-response or moderate OHSS. Again, the major disadvantage of kisspeptin trigger is the cost of the preparation.

### hCG versus GnRHa trigger

When comparing the effect of hCG versus GnRHa trigger on the different follicular maturation variables following an IVF treatment cycle, studies have revealed that the number of oocytes retrieved, percentage of mature oocytes, and number of top-quality embryos were either comparable or in favor of the GnRHa trigger. Moreover, studies of the downstream effects of LH receptor activation by LH or hCG have demonstrated that LH has a greater impact on AKT and ERK1/2 phosphorylation (responsible for proliferation, differentiation, and survival of GCs), while hCG generates higher intracellular cAMP accumulation, which stimulates steroidogenesis (progesterone production) [12, 13].

Moreover, human mural GCs gene expression related to steroidogenesis (*StAR/CYP19)* and oocyte maturation (*COX2/Amphiregulin)* in cultured GCs have demonstrated that the addition of LH + FSH + hCG showed higher activation of steroidogenesis and maturation as compared to the naturally occurring trigger (LH + FSH) and the hCG triggers [14]. Moreover, while the naturally occurring trigger (LH + FSH) activated maturation significantly and more intensely than the hCG trigger, no between-group differences were observed with regard to steroidogenic-related genes.

Based on these observations, GnRHa, combined with hCG trigger, for final follicular maturation has been implemented in clinical practice. Modes and timing of administration should be appropriately tailored to various subgroups of IVF patients.

## Dual-trigger standard hCG dose concomitant with GnRHa, 35–37 h before oocyte retrieval

Research findings demonstrating comparable or even improved maturity and quality of oocytes/embryos following GnRHa trigger, as compared to hCG trigger, and the different effects of LH and hCG on the downstream signaling of the LH receptor, have led to a new strategy for final follicular maturation: concomitant administration of both GnRHa and a standard bolus of hCG (5000–10,000 units) prior to oocyte retrieval. This approach aims to improve oocyte and embryo quality, to optimize the ultimate outcome of the IVF cycle.

Lin et al. [15], in their retrospective cohort study, compared IVF outcome in normal-responder patients undergoing COH using GnRHa with either a standard dose of hCG trigger (6500 IU recombinant hCG) or the dual trigger (0.2 mg triptorelin + 6500 IU recombinant hCG) 35–36 h prior to oocyte retrieval. The dual-trigger group demonstrated a statistically significantly higher number of oocytes retrieved, matured oocytes, and number of embryos cryopreserved; this resulted in a significant increase in implantation, clinical pregnancy, and live-birth rates compared with the hCG-only trigger group.

In a subsequent prospective randomized controlled trial of normal-responder patients, Decleer et al. [16] compared IVF outcome following either 5000 IU hCG trigger or a combination of GnRHa plus 5000 IU hCG concomitantly, 36 h prior to oocyte retrieval. Although no between-group differences were observed in the mean number of oocytes retrieved, mature oocytes, or pregnancy rates, the number of patients who received at least one embryo of excellent quality and the number of cryopreserved embryos were significantly higher following the dual trigger.

In a single-center randomized study (155 normal responders), using the dual trigger for final follicular maturation, has demonstrated an increased number of oocytes, mature oocytes, and number of blastocysts (total and top-quality) compared with hCG alone. Clinical pregnancies (46.1% vs. 24.3%) and cumulative delivery rate (36.2% vs. 22%) were significantly higher in the dual-trigger group [17].

## Double trigger- GnRHa 40 h and standard hCG added 34 h prior to OPU, respectively

Beck-Fruchter et al. [18] have described a case of recurrent empty follicle syndrome, successfully treated by ovulation trigger with GnRHa and hCG added 40 h and 34 h, respectively, before oocyte retrieval. They assumed that by prolonging the time between ovulation triggering and OPU [19] and the GnRHa trigger with the consequent simultaneous induction of an FSH surge, the “double trigger” could overcome any existing impairments in GC function, oocyte meiotic maturation, or cumulus expansion, resulting in successful aspiration of mature oocytes, pregnancy, and delivery.

Encouraged by these findings, we offered the double-trigger approach to two groups of patients showing abnormal final follicular maturation despite normal response to COH: those with < 50% oocytes retrieved per number of dominant follicles > 14 mm in diameter on day of hCG administration [20], and those with < 66% mature/metaphase-II (MII) oocytes per number of oocytes retrieved [21].

In the group with < 50% oocytes retrieved per number of dominant follicles, following the double trigger, patients had a significantly higher number of oocytes retrieved, number of 2PN, and number of embryos transferred, and significantly higher proportions of number of oocytes retrieved to number of follicles > 10 and > 14 mm in diameter on day of hCG administration, with a tendency toward a higher number of top-quality embryos, compared with the hCG-only trigger cycles [20].

In the group with < 66% MII oocytes per number of oocytes retrieved, following the double trigger, patients yielded a significantly higher number of MII oocytes and proportion of MII oocytes per number of oocytes retrieved, resulting in a significantly increased number of top-quality embryos, compared with the hCG-only trigger cycles [21].

## Standard hCG dose concomitant with GnRHa (dual trigger), 34 h before OPU

According to the Bologna criteria [22], the minimal criteria needed to define poor ovarian response (POR) are the presence of at least two of the following three features:

- Advanced maternal age (≥ 40 years) or any other risk factor for POR.
- A previous POR (≤ 3 oocytes with a conventional stimulation protocol).
- An abnormal ovarian reserve test.

One of the major unnoticed concern in this group of poor responders is the observed high prevalence of premature luteinization/ovulation [23, 24], which may be overcome by early triggering of final follicular maturation (when approaching a follicular size of 15–16 mm) and by shortening the duration between the trigger and OPU. However, since shortening the interval between hCG priming and oocyte retrieval can decrease the percentage of mature oocytes [25], an additional measure should be implemented to improve the number of oocytes retrieved to the number of follicles > 10 mm, as well as to increase the proportion of mature oocytes. Suggested measures to achieve this are to dual trigger 34 h before OPU when the leading follicle reaches > 17 mm in diameter, or to double trigger when the leading follicle reaches < 17 mm in diameter on the day of trigger [25, 26].

## Conclusions

**Fig. 2:**
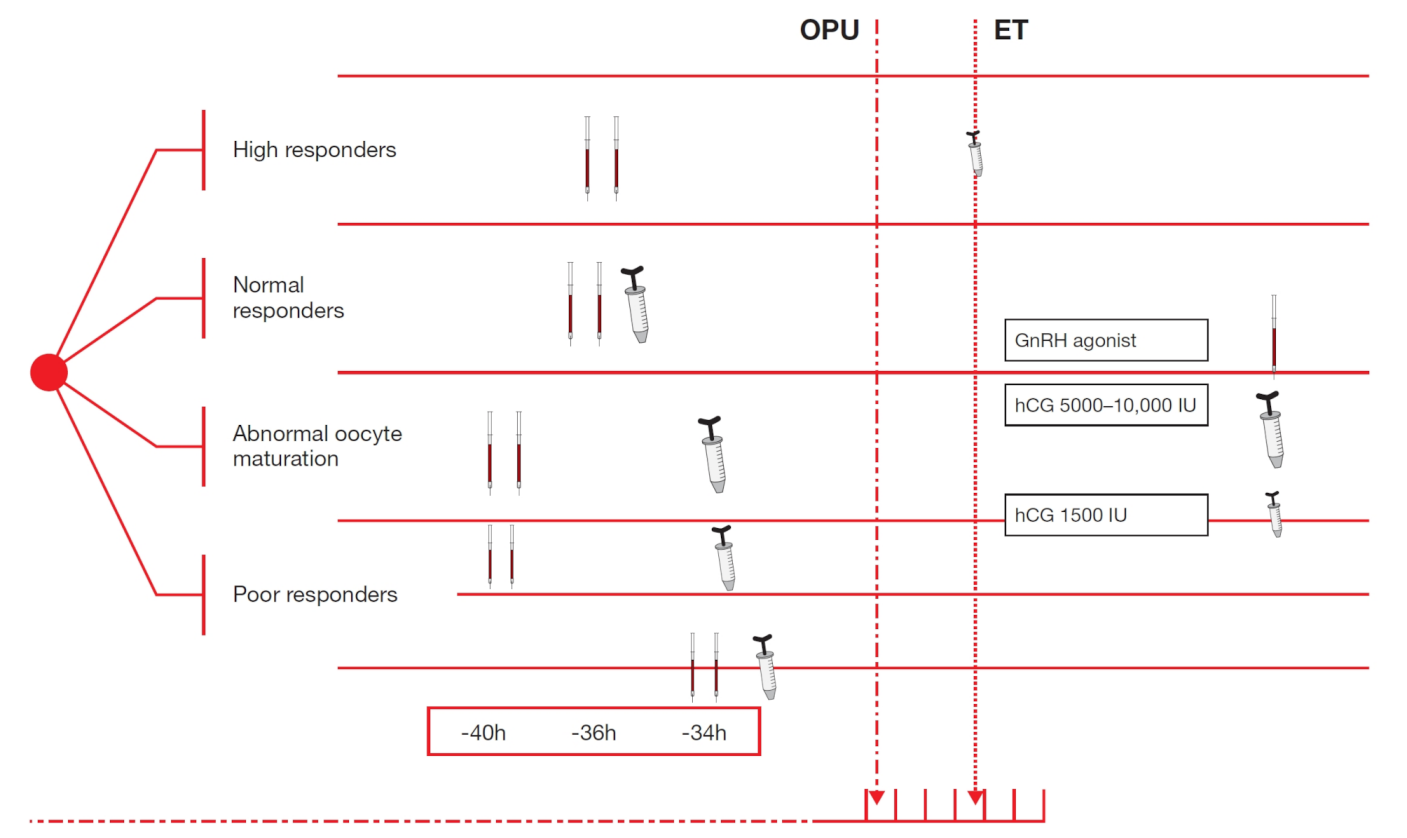
Triggering final follicular maturation

This review explores published studies relating to various means of triggering final follicular maturation, indicating how to tailor each approach for specific patient subgroups (Fig. 2). We believe that adopting appropriately tailored trigger strategies has the potential to improve IVF outcomes for patients [26].

## Data Availability

Not applicable.

## Abbreviations

COH: controlled ovarian hyperstimulation
FSH: follicle-stimulating hormone
GC: granulosa cell
GnRH[a]: gonadotropin-releasing hormone [agonist
hCG: human chorionic gonadotropin
IVF: in-vitro fertilization
LH: luteinizing hormone
MII: mature/metaphase-II
OHSS: ovarian hyperstimulation syndrome
OPU: oocyte pick-up
POR: poor ovarian response
rhLH: recombinant human luteinizing hormone
