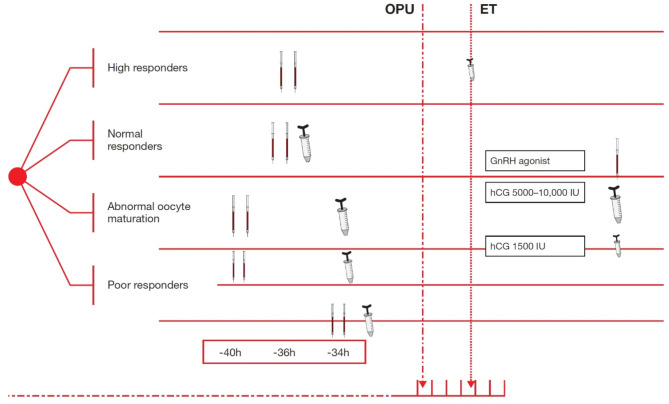# Correction: Triggering final follicular maturation for IVF cycles

**DOI:** 10.1186/s12958-025-01363-6

**Published:** 2025-02-20

**Authors:** Raoul Orvieto

**Affiliations:** 1 Infertility and IVF Unit, Department of Obstetrics and Gynecology, Chaim Sheba Medical Center (Tel Hashomer), Ramat Gan, 52621 Israel; 2 Israel and the Tarnesby-Tarnowski Chair for Family Planning and Fertility Regulation, Faculty of Medical and Health Science, Tel-Aviv University, Tel Aviv, Israel

**Correction: Reproductive Biology and Endocrinology (2025) 23:12**

10.1186/s12958-024-01332-5

After publication of the article [1], it was brought to the journal that Figs. 1 and 2 were not updated.

The incorrect Fig. 1:

**Figure:**
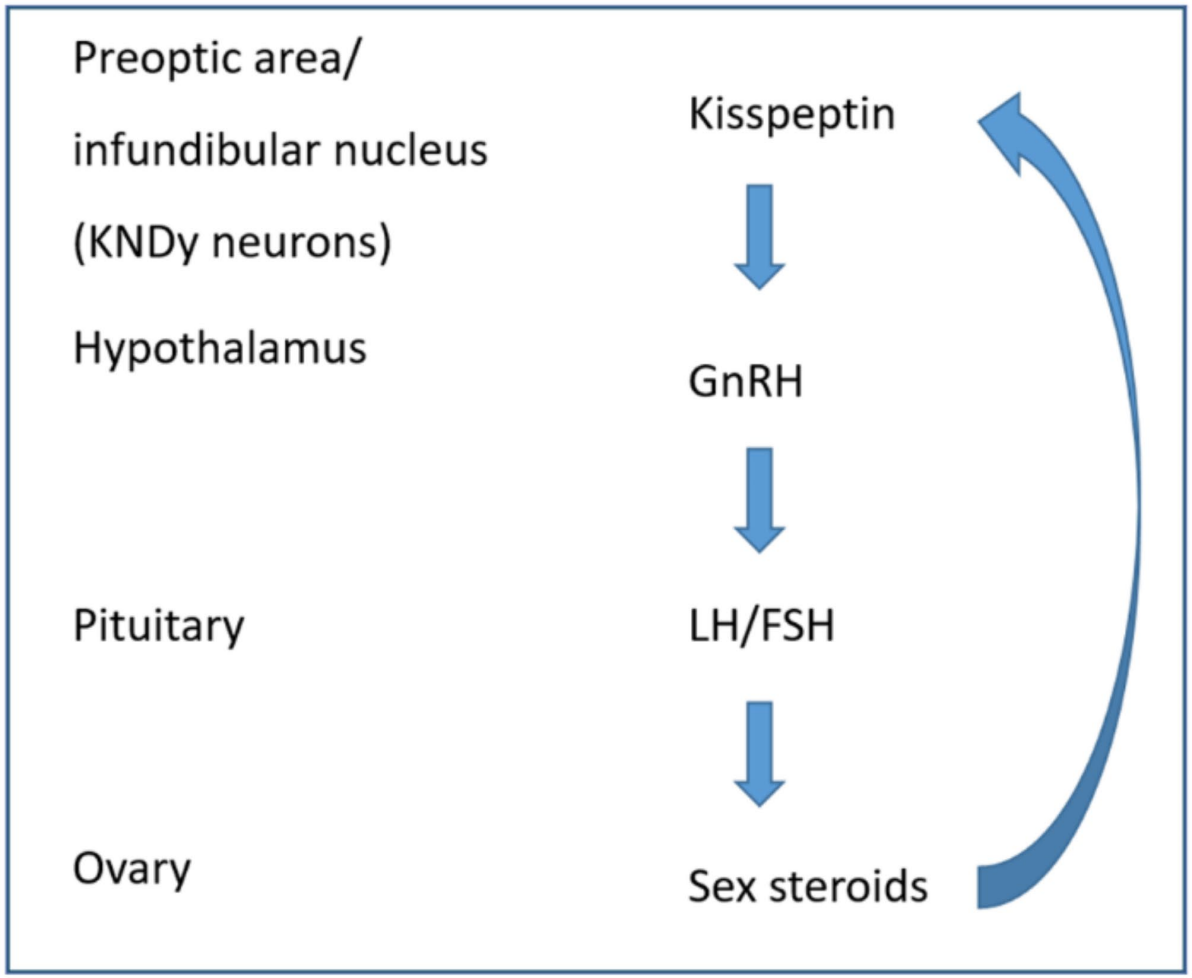

The incorrect Fig. 2:

**Figure:**
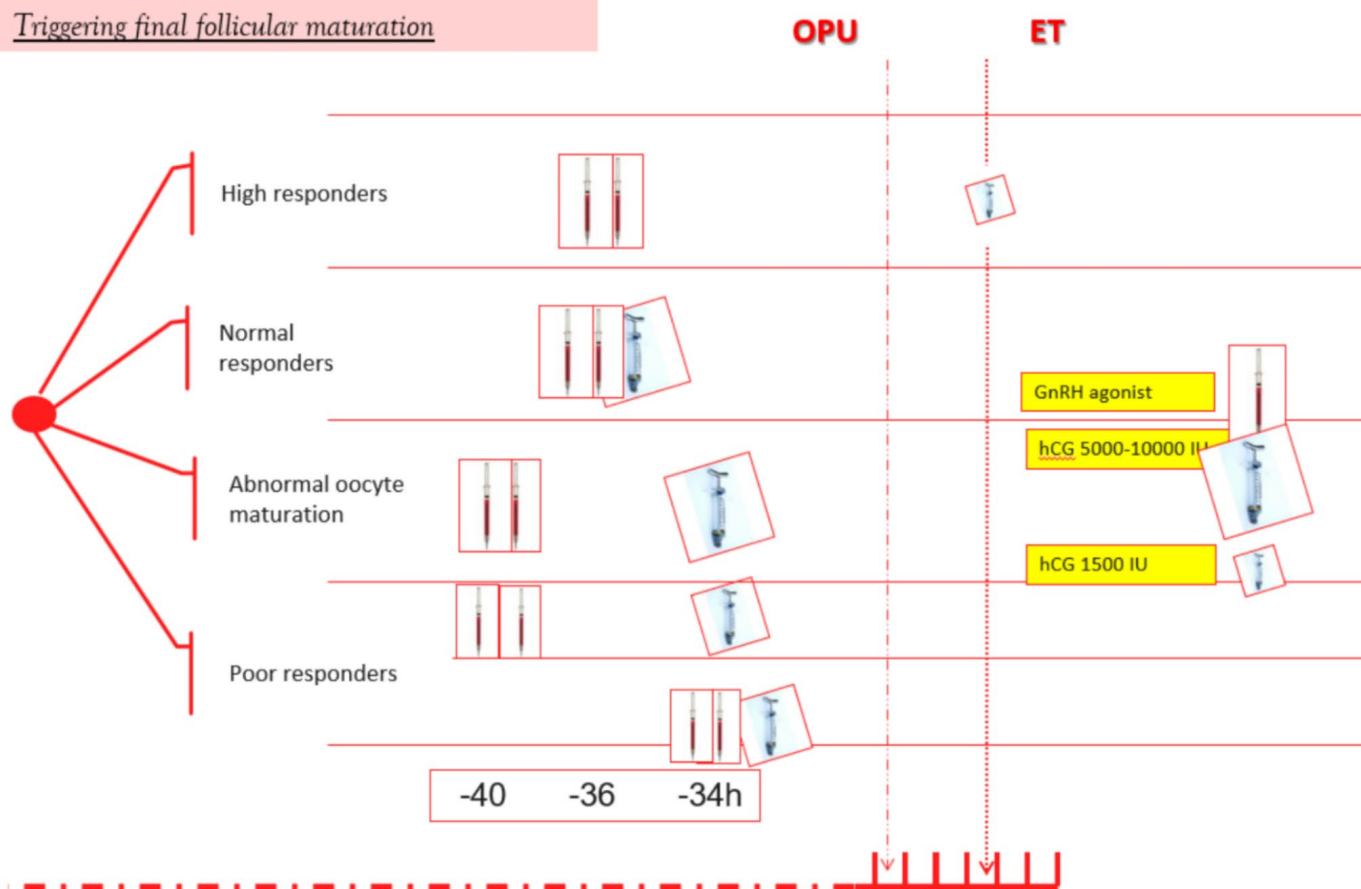

The correct Fig. 1:

**Figure:**
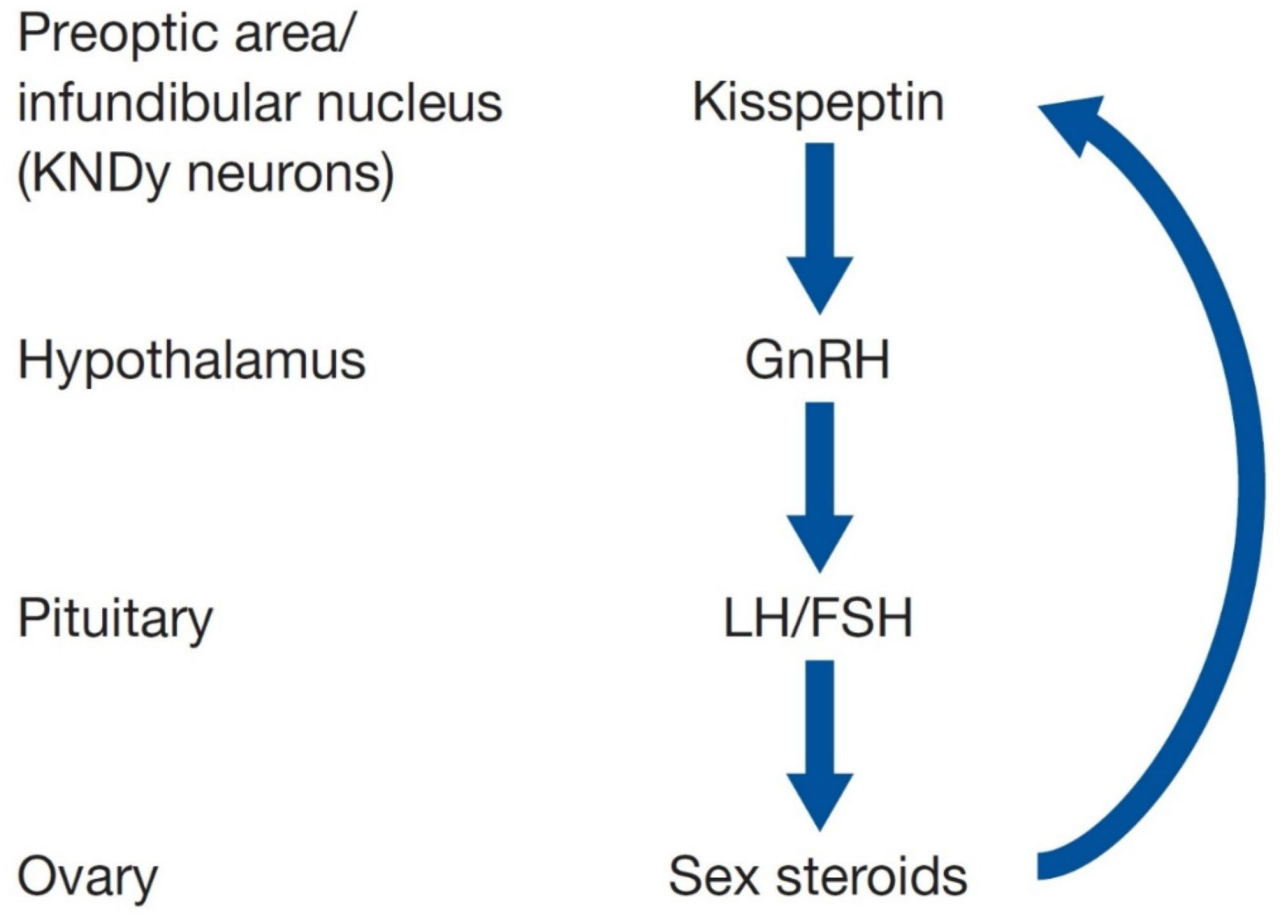

The correct Fig. 2:

**Figure:**